# State-of the-art and future perspective in co-culture systems for tendon engineering

**DOI:** 10.1016/j.bbiosy.2025.100110

**Published:** 2025-03-05

**Authors:** Salomé Guillaumin, Andrea Rossoni, Dimitrios Zeugolis

**Affiliations:** a Regenerative, Modular & Developmental Engineering Laboratory (REMODEL) and Science Foundation Ireland (SFI) Centre for Research in Medical Devices (CÚRAM), Biomedical Sciences Building, University of Galway, Galway, Ireland; b Regenerative, Modular & Developmental Engineering Laboratory (REMODEL), Charles Institute of Dermatology, Conway Institute of Biomolecular and Biomedical Research and School of Mechanical and Materials Engineering, University College Dublin (UCD), Dublin, Ireland

**Keywords:** Tenocytes, Mesenchymal stromal cells, Co-culture, Tissue engineering, Regenerative medicine

## Abstract

Tendon is a connective tissue that links bone to muscle, allowing for maintenance of skeleton posture, joint movement, energy storage and transmission of muscle force to bone. Tendon is a hypocellular and hypovascular tissue of poor self-regeneration capacity. Current surgical treatments are of limited success, frequently resulting in reinjury. Upcoming cell therapies are primarily based on tenocytes, a cell population of limited self-renewal capacity in vitro or mesenchymal stromal cells, a cell population prone to ectopic bone formation in vivo. Over the years mono- or multi- factorial cell culture technologies have failed to effectively maintain tenocyte phenotype in culture during expansion or to prime mesenchymal stromal cells towards tenogenic lineage prior to implantation. Upon these limitations the concept of co-culture was conceived. Here, we comprehensively review and discuss tenogenic differentiation of mesenchymal stromal cells through direct or indirect culture with tenocytes in an attempt to generate a tenocyte or a tendon-like cell population for regenerative medicine purposes.

## Highlights

• In tendon engineering, tenocytes and mesenchymal stromal cells are the most studied cell populations.
• Tenocytes rapidly lose their phenotype and function in vitro, whilst naïve mesenchymal stromal cells are often associated with ectopic bone formation in vivo.
• Priming mesenchymal stromal cells towards tenogenic lineage via co-culture with tenocytes may be a valuable alternative.
• Data obtained to-date clearly demonstrate the potential of the approach, but detailed investigations are needed.

## 1 Introduction

**Fig. 1:**
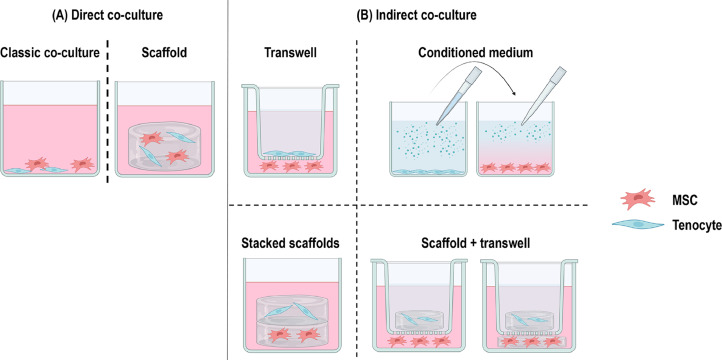
Co-culture strategies based on tenocytes and MSCs in tendon engineering.

Co-culture methods are long-established techniques that allow the study of direct and / or indirect interactions between different cell types cultured in a shared environment [1]. Co-culture systems can be either direct (Fig. 1**a**) or indirect (Fig. 1**b**). In the direct co-culture, the different cell types are in contact with each other, thus allowing paracrine signalling and physical cell-cell communication [2,3]. In the indirect co-culture, the different cell types are separated by a permeable barrier, thus allowing only paracrine signalling [4]. Indirect co-culture can be further limited to one-way communication by culturing one cell population with secretome, in the form of conditioned medium (CM), from another cell population [5]. Considering that in vivo multiple cell types are in direct contact or in close proximity and this dynamic reciprocity determines cell / tissue function [[6], [7], [8]], co-culture systems are extensively used, among others, to study physiological processes [[9], [10], [11], [12], [13]]; disease manifestation, progression and treatment modalities [[14], [15], [16], [17]]; and reparative therapies [[18], [19], [20]].

The utilisation of co-culture techniques to induce mesenchymal stromal cells (MSCs) lineage-specific differentiation (e.g. chondrogenic [[21], [22], [23]], osteogenic [[24], [25], [26]], cardiac [[27], [28], [29]], neural [[30], [31], [32]] differentiation) is a compelling area of research within the field of tissue engineering. Through co-culture, functional tissue-specific cell populations can be developed for tissues of low cellularity and cells of reduced function and / or limited expansion capacity *ex vivo*. The distinct advantages of co-culture systems are two-fold. Firstly, genetic engineering approaches that may prolong or even prohibit clinical translation and commercialisation are avoided. Secondly, considering the infinite number of permutations (e.g. molecular options, doses, timing, sequence, etc.), it is unlikely that we will ever be able to develop cell culture media with appropriate supplements that will be as effective in MSC differentiation as the secretome of the targeted tissue cells.

With these in mind, herein we critically discuss tenogenic differentiation of MSCs through tenocyte (TC) co-culture approaches. To put our study into context, we first describe the clinical need, followed by the characteristics of the two protagonists (i.e. the MSCs and the TCs). We also discuss preclinical data to inform future clinical trials in this direction.

## 2 The clinical need

Regeneration of injured tendons remains a clinical challenge. Delayed diagnosis and treatment of tendinopathy often leads to tissue degeneration, which is associated with severe pain and impaired function [33,34], ultimately leading to rupture [35], sometimes without any warning [36]. Symptomatic tendinopathies account for one-third of all the musculoskeletal diseases in US, with a total annual healthcare cost of US$ 380.9 billion [37,38]. It is also worth noting that tendon injuries are associated with reduced quality of life, productivity loss and high indirect costs due to worker's compensation for disease [39]. Current treatments for tendon injuries reside in conservative (e.g. functional rehabilitation, cast immobilisation and anti-inflammatory drugs [40,41]) or surgical (e.g. suturing, tissue grafts and biomaterials [[42], [43], [44]]) methods. Unfortunately, such approaches only partially restore structural, mechanical and functional properties [45,46]; these precise limitations formed the foundations for developing cell-based therapies for tendon repair and regeneration [47]. Among the various cell populations available, the most prominent players in vitro and in preclinical settings have been TCs and MSCs [47,48]. Interestingly though, to-date, only a few studies (either registered at ClinicalTrials.Gov or available in PubMed) have assessed in clinical setting either of these cells in a tendon injury / pathophysiology context (Table 1). Although overall the results are positive, not one multicentre, randomised, triple-blinded and controlled clinical trial has been conducted to-date. With respect to TCs, we believe that this limited clinical assessment is due to their low availability and their rapid phenotypic drift and loss of functionality during in vitro culture [49,50]. MSCs have their own share of limitations, the major one being the possibility of heterotopic ossification [51] that puts forward the notion of priming MSCs towards tenogenic differentiation prior to implantation. Taking these into consideration, at this point, it is important to meet / define our protagonists, the TCs and the MSCs.

**Table 1:** Active [Source: ClinicalTrials.Gov; Terms searched: tendon in ‘condition / disease’ and stem cell(s) (5 studies) or tenocyte(s) (0 studies) or tendon fibroblast(s) (0 studies) or stromal vascular fraction (3 studies) in ‘intervention / treatment’ with the ‘not yet recruiting’, ‘recruiting’ and ‘active, not recruiting’ ticked; Date of search: 02/02/2024] and completed [Source: PubMed; Terms searched: tendon and tenocyte(s) or tendon fibroblast(s) or stem cell(s), in Title / Abstract with clinical trials ticked; Date of search: 01/02/2024)] clinical studies using human TCs or human MSCs in tendon engineering context.

Study description	Comments
A prospective randomised trial of biologic augmentation with mesenchymal stromal cells in patients undergoing arthroscopic rotator cuff repair.	NCT02484950 (active, not recruiting)
A prospective, double-blinded, multi-centre pivotal trial of autologous adult adipose-derived regenerative cell injection into chronic partial-thickness rotator cuff tears.	NCT03752827 (active, not recruiting)
Effectiveness of stromal vascular fraction and platelets rich plasma in osteoarthritis and tendinopathy: Study protocol for a phase III, prospective, randomized, controlled multi-centre study.	NCT05660824 (not yet recruiting)
A prospective, randomised with one arm study aiming to determine the efficacy of an amniotic fluid tissue product for pain relief and functional improvements for different types of musculoskeletal conditions.	NCT03390920 (not yet recruiting)
A two-arm, double-blinded, randomised controlled trial aiming to determine if the effectiveness of a single injection of connective tissue matrix boost in the treatment of patients with rotator cuff tendinopathy is as effective as a single injection of platelet rich plasma in reducing the symptoms of rotator cuff tendinopathy.	NCT06160427 (not yet recruiting)
An interventional pilot study aiming to evaluate clinical and radiological results after treatment of patellar tendinopathy with injected autologous ultrasound-guided, intra- and peri-tendon stromal vascular fraction.	NCT04753853 (active, not recruiting)
A phase II placebo and randomised study with quadruple masking (participant, care provider, investigator, outcomes assessor) aiming to evaluate autologous stromal vascular fraction cell therapy to improve the repair of chronically rotator cuff tears.	NCT03332238 (active, not recruiting)
A multi-centric, randomized, triple-blinded controlled trial aiming to assess the effectiveness of stromal vascular fraction and platelets rich plasma in osteoarthritis and tendinopathy	NCT05660824 (active, not recruiting)
To assess the safety and efficacy of OrthADAPT™ (collagen type I membrane) or OrthADAPT™ and autologous BM-MSCs for repairing full thickness rotator cuff tears. A randomised, double-blinded and placebo-controlled trial.	Adverse effects in both groups resulted in trial termination. Data obtained illustrate the need to either use a different scaffold or to not use a scaffold for small joints [92].
To assess safety and efficacy of fibrin glue delivery of allogeneic AD-MSCs in the treatment of lateral epicondylosis. 12 participants (6 received 1000,000 cells and 6 received 10,000,000 cells).	No significant adverse effects were observed through the 52 weeks of follow-up. Progressively, the patients’ visual analogue scale scores were decreased, the elbow performance scores were increased and the tendon defects were decreased [93].
To investigate the effect of allogenic AD-MSCs in fibrin glue, saline / fibrin glue mixture and saline only injections in the treatment of partial tears in the supraspinatus tendon. 24 participants (8 in each group) with 2 years follow up period. A randomised and controlled trial.	No differences were observed between the groups in improvement of pain, shoulder function, lesion size and adverse events [94].
To assess the safety and efficacy of single-row arthroscopic rotator cuff repair without adipose tissue injection and single-row arthroscopic rotator cuff repair followed by intraoperative injection of autologous micro-fragmented adipose tissue processed with an enzyme-free technology in arthroscopic rotator cuff repair. 177 participants were screened and 44 (22 in each group) completed a 2 year follow up period. A randomised, single-blinded and controlled trial.	Although the intraoperative injection of autologous micro-fragmented adipose tissue was found to be safe and effective in improving short-term clinical and functional outcomes, no significant differences were observed between the groups in re-rupture rate, complication rate, number of adverse events and mid-term clinical outcomes [95].
35 participants received arthroscopic rotator cuff repair alone and 35 matched (based on sex, age and lesion size) participants received arthroscopic rotator cuff repair with an injection of autologous AD-MSCs loaded in fibrin glue. 28 month follow up period.	Significant improvement of structural outcomes in terms of retear rate were achieved through the use of AD-MSCs, but no significant differences between the groups were observed in the mean visual analogue scale; in Constant score and University of California Los Angeles shoulder rating scale; and in range of motion (including forward flexion, external rotation at the side and internal rotation at the back) measures [96].
A flexor digitorum profundus tendon zone I rupture of a sport climbing athlete was repaired using an autologous palmaris longus tendon graft, an allogeneic amniotic membrane and allogeneic AD-MSCs. 3 years follow up period.	The athlete was able to resume competitive climbing due to nearly normal tissue function and gliding [97].
To compare the efficacy of platelet-rich plasma (23 participants) and stromal vascular fraction (21 patients) injections for the treatment of non-insertional Achilles tendinopathy. Participants were treated unilaterally or bilaterally for a total of 28 tendons per group. Randomised, double blinded and controlled trial. 6 month follow up period.	Although at 15 and 30 days the stromal vascular fraction group outperformed the platelet-rich plasma group, as judged by visual analogue scale, American Orthopaedic Foot and Ankle Society and Victorian Institute of Sport Assessment-Achilles scores, at the subsequent time points (2, 4 and 6 months), the scores were not significantly different between the groups [98].
Autologous patellar tendon TC injection into the extensor carpi radialis brevis tendon for the treatment of severe, chronic resistant lateral epicondylitis. 18 participants with 12 months follow up period.	Improved mean visual analogue scale pain score, decreased pain score at 12 months; improved mean quick disabilities of the arm, shoulder and hand and grip strength scores; and improved grade of tendinopathy at the common extensor origin [99].
Autologous patellar tendon TC injection into the extensor carpi radialis brevis tendon for the treatment of severe, chronic resistant lateral epicondylitis. 15 participants with mean 4.5 years follow up period.	Improved mean visual analogue scale pain; quick disabilities of the arm, shoulder and hand scores; upper extremity functional scale score; grip strength; and MRI tendinopathy score [100].
Autologous palmaris longus TC injection into the subscapularis site of an elite swimmer. 4 months follow up period.	Three blinded independent radiologist MRI review demonstrated a significant reduction in tear size and improved tendon morphology. Internal rotation strength returned to baseline strength levels after 6 weeks and the athlete returned to full training and international level competition without pain after 4 months [101].
To assess the safety and effectiveness of autologous patella tendon TC injection in patients with chronic recalcitrant gluteal tendinopathy. 12 participants for 24 months follow up period.	The Oxford hip score, the visual analogue pain scale score and the short form-36 were significantly improved up to 12 months and remained constant until month 24. From 12 participants, 8 were satisfied with the outcomes. Significant MRI improvement was not demonstrated [102].
To compare BM-MSCs and leukocyte-poor platelet-rich plasma effectiveness on patellar tendon structure and regeneration. 20 patients with recalcitrant patellar tendinopathy for 12 months follow up period. Patients demonstrating no patellar tendon regeneration with leukocyte-poor platelet-rich plasma treatment at the 6 months follow up were offered BM-MSC treatment and were followed for another 6 months follow up period.	The tendon morphology, assessed by MRI scan, the visual analogue pain scale and Victorian Institute of Sport Assessment-Patella scores were significantly improved up to 12 months in BM-MSC group. Significant reduction of lesion size was also observed. Similarly, tendon morphology and Victorian Institute of Sport Assessment-Patella score were significantly improved up to 12 months in leukocyte-poor platelet-rich plasma group followed by BM-MSC treatment, however, visual analogue pain scale score during sport and lesion size did not show statistical difference. No untreated control group [103].

Abbreviations: AD-MSCs: adipose derived mesenchymal stromal cells, BM-MSCs: bone marrow mesenchymal stromal cells, MRI: magnetic resonance imaging, TC: tenocyte

## 3 TCs and MSCs

Back in 2006, in a position statement, the International Society for Cellular Therapy (ISCT) identified three minimal traits to define MSCs: plastic-adherent in normal cultures; positive for CD105, CD73 and CD90 and negative for CD45, CD34, CD14 or CD11b, CD79alpha or CD19 and HLA-DR surface molecules; and able to differentiate in vitro towards osteogenic, adipogenic and chondrogenic lineages cells [52]. Since then, the list has been expanded to include a number of phenotypic (e.g. CD29, CD44, CD146, CD166, CD271, CD36, CD163, CD140b, CD200, CD248, CD273, CD274, STRO-1, MSCA-1, SSEA-4) and impurity (e.g. CD3, CD13, CD31, CD133) markers [[53], [54], [55], [56]]. Table 2 provides a comprehensive list of human MSC markers, along with their function and human MSC population identified.

**Table 2:** Human MSC markers and function commonly studied via flow cytometry.

Marker and MSC	Function
**CD11b-** BM-MSCs [104], AD-MSCs [105], UCB-MSCs [104]	CD11b is an integrin which is coupled with CD18 to form the complement receptor type 3, transmembrane receptor found on most human immune cells. It has a phagocytic [106] and adhesion [107] role in human immune cells.
**CD13±** BM-MSCs [108], AD-MSCs [109], SF-MSCs [108], AM-MSCs [110], CH-MSCs [110], G-MSCs [111]	CD13 is a metalloprotease found in myeloid cells and is necessary in human umbilical vein endothelial cell capillary formation [112]. In hBM-MSCs, CD13 acts as signal transducing adhesion molecule to mediate their adhesion, migration and invasion [113].
**CD14-** BM-MSCs [104], AD-MSCs [114], UCB-MSCs [104], SF-MSCs [115], AM-MSCs [110], CH-MSCs [110], G-MSCs [111]	CD14 is a glycolipid-anchored membrane glycoprotein expressed on human monocyte and macrophage surfaces [116]. It is used as a pattern recognition receptor in innate immunity for several ligands, including but not limited to apoptotic cells [117] and bacterial products [118].
**CD19-** BM-MSCs [104], AD-MSCs [104], UCB-MSCs [119]	CD19 is a type I transmembrane protein that affects human B cell activation through toll like receptor [120]. Mutation of CD19 leads to defective response from human B cells to antigenic stimulation, which results in a poor reaction to vaccination [121].
**CD29±** BM-MSCs [104], AD-MSCs [122], UCB-MSCs [123], AM-MSCs [110], CH-MSCs [110], WJ-MSCs [124], P-MSCs [125], G-MSCs [111]	CD29 is an integrin β1 which, when present on MSCs (rat BM-MSCs and AD-MSCs), was associated with reduced gene expression of stem cell markers and reduced capacity for osteogenic and adipogenic differentiation [126]. Inhibition of CD29 led to reduced rat BM-MSC migration [127]. Very late antigen-4 is an integrin dimer composed of CD49d and CD29. Very late antigen–4 is associated to hBM-MSC adherence on human endothelial cells [128].
**CD29-** UCB- MSCs [119]	
**CD31-** BM-MSCs [129], AD-MSCs [130], UCB-MSCs [119], UC-MSCs [129], P-MSCs [125], G-MSCs [111]	CD31 is a transmembrane glycoprotein, also known as platelet / endothelial cell adhesion molecule-1, is present on human platelet and endothelial cell surface [131] and is required for human leukocyte transendothelial migration, where its localisation is at the endothelial junctions [132].
**CD34±** AD-MSCs [114]	CD34 is a membrane protein expressed on human hematopoietic cells [133]. CD34+ hAD-MSCs appeared to have increased proliferation capacity compared to CD34- hAD-MSCs yet, CD34- hAD-MSCs demonstrated higher osteogenic and adipogenic differentiations compared to the CD34+ cells [134]. CD34+ mouse BM-MSCs have shown greater angiogenesis and vasculogenesis capacities than CD34- mouse BM-MSCs [135].
**CD34-** BM-MSCs [104], AD-MSCs [104], UCB-MSCs [104], SF-MSCs [115], AM-MSCs [110], CH-MSCs [110], WJ-MSCs [124], G-MSCs [111]	
**CD36-** BM-MSCs (not expressed in one donor and expressed in another donor) [136], BM-MSCs [53], AD-MSCs [136], UCB-MSCs [136]	CD36 is a cell surface protein belonging to the scavenger receptor class B family [137] and was identified first as a human platelet membrane glycoprotein [138]. CD36 is a receptor for thrombospondin-1 [139]. CD36 is involved in human platelet adhesion [140] and in bacterial recognition and phagocytosis [141].
**CD44±** BM-MSCs [104], AD-MSCs [109], UCB-MSCs [104], SF-MSCs [115], AM-MSCs [110], CH-MSCs [110], WJ-MSCs [124], P-MSCs [125], G-MSCs [111]	CD44 is an adhesion molecule that interacts with hyaluronan and is expressed by hBM-MSCs during culture but not in vivo [142]. CD44- hBM-MSCs are more quiescent than their CD44+ counterparts [142]. CD44 expression on hWJ-MSC may help them maintaining stemness phenotype [143]. Inhibition of CD44 in hMSCs (tissue was not specified) decreased hMSC migration [144].
**CD45-** BM-MSCs [129], AD-MSCs [114], UCB-MSCs [129], UC-MSCs [129], SF-MSCs [115], AM-MSCs [110], CH-MSCs [110], WJ-MSCs [124], P-MSCs [125], G-MSCs [111]	CD45 is a membrane glycoprotein expressed by almost all human hematopoietic cells except mature erythrocytes. CD45 role in human hematopoietic cells lies on cell activation and differentiation [145].
**CD51±** AD-MSCs [105]	CD51 is an integrin α5. Platelet derived growth factor α+ / CD51+ human and mouse BM-MSCs expressed high protein levels of hematopoietic stem cell markers [146]. CD51+ / CD271+ hBM-MSCs appeared to have superior chondrogenic differentiation potential compared to CD51- / CD271- hBM-MSCs [147].
**CD51-** BM-MSCs [146], UCB-MSCs [119]	
**CD54±** AD-MSCs [114], SF-MSCs [148], AM-MSCs [110], CH-MSCs [110]	CD54 is a cell surface glycoprotein which mediate human endothelial cell and T cell interactions [149]. CD54+ hBM-MSCs exerted an immune-suppressive effect by acting on pro-inflammatory macrophages, which happened through CD54 and led to an inhibition of human T cell proliferation [150].
**CD54-** BM-MSCs [130], AD-MSCs [114]	
**CD55±** BM-MSCs [114], AD-MSCs [114], UC-MSCs [151], SF-MSCs [108]	CD55 is a cell surface glycoprotein and has a role of inhibitor of the complement, which was found to be upregulated in hAD-MSCs compared to hBM-MSCs and hUC-MSCs. In vivo (mouse), hAD-MSCs demonstrated higher survival than hBM-MSCs and hUC-MSCs. Therefore, CD55 offered protection against complement-mediated injury in a graft versus host disease environment upon cell transplantation. Using atorvastatin or erlotinib, it is possible to increase gene and protein CD55 expression on hBM-MSCs, hAD-MSCs and hUC-MSCs [151].
**CD56-** BM-MSCs, UCB-MSCs, UC-MSCs [129]	CD56 is a cell surface glycoprotein mainly expressed in human natural killer cells. CD56+ human T cells demonstrated to proliferate less and have an enhanced major histocompatibility complex cytotoxicity activity coupled with higher IL-3 and IFN-γ release upon CD3 activation compared with CD56- human T cells [152].
**CD59±** BM-MSCs [114], AD-MSCs [122]	CD59 is a cell-surface glycoprotein. Its over-expression in human liver MSCs by the mean of recombinant vectors allowed for protection from complement-mediated lysis, therefore permitting for complement evasion [153]. In contrast with this study, a paper introduced CD59 as an activator of the immune system, which should be down-regulated using cannabidiol on hG-MSCs [111].
**CD73±** BM-MSCs [154], AD-MSCs [109], UCB-MSCs [123], UC-MSCs [129], SF-MSCs [115], AM-MSCs [110], CH-MSCs [110], WJ-MSCs [124], G-MSCs [111]	CD73 is a 5′nucleotidase that rules the hydroxylation of adenosine monophosphate to adenosine in the extracellular space [155]. CD73+ mouse AD-MSCs demonstrate superior myocardial regeneration compared to unsorted mouse AD-MSCs, due to increased angiogenesis [156]. Also, the superior regeneration capacity of CD73+ mouse pericardial AD-MSCs, compared to phosphate buffer solution control and CD73- mouse pericardial AD-MSCs, is due to anti-inflammatory activity, by attenuating C-C chemokine receptor 2 macrophage infiltration and upregulating anti-inflammatory genes [157]. CD73+ mouse BM-MSCs displayed higher osteogenic capacity, compared to CD73- counterparts [158].
**CD79a-** BM-MSCs, AD-MSCs and UCB-MSCs [104]	CD79a is a B lymphocyte antigen receptor present in both normal and neoplastic human B cells. CD79a is expressed early in human B cell maturation and is present in most leukemias and lymphomas [159].
**CD90±** BM-MSCs [123], AD-MSCs [109], UCB-MSCs [123], UC-MSCs [129], SF-MSCs [115], AM-MSCs [110], CH-MSCs [110], G-MSCs [111]	CD90 is a glycoprotein anchored to the cell surface. Upon adipogenic differentiation, a decrease in CD90 gene expression in hUCB-MSCs was observed [160]. Similarly, a decrease of CD90 using a CD90-target small hairpin RNA lentiviral vectors in multiple hMSC sources (i.e. adipose, dental pulp and amniotic fluid) led to an increase in adipogenic and osteogenic differentiations and a decrease in protein expression of CD44 and CD166 (i.e. MSC markers) [161].
**CD90-** UCB-MSCs [119], WJ-MSCs [124]	
**CD105±** BM-MSCs [162], AD-MSCs [109], UCB-MSCs [104], UC-MSCs [129], SF-MSCs [115], AM-MSCs [110], CH-MSCs [110], G-MSCs [111]	CD105 is a transmembrane protein. Its presence on hBM-MSCs was suspected to enhance chondrogenesis [162]. This hypothesis was confirmed on hAD-MSCs [163], but another report related contrasting results [164]. hWJ-MSC expressing CD105 have superior osteogenic potential [143]. CD105 depletion in hAD-MSCs enhanced osteogenic differentiation [165]. CD105 expression in hUCB-MSCs was associated to improved myocardial regeneration in mouse, effect accounted to proliferation or cytoprotection under hypoxic conditions from CD105+ human cells [166]. Lack of CD105 marker on hAD-MSCs and hUC-MSCs was associated to higher immunomodulation capacities, with a decrease in T lymphocyte proliferation along with reduced cytokine (i.e. IFN-γ, TNF-α, IL-1β and IL-2) secretion [167]. CD105 is lost upon hUC-MSC differentiation, suggesting its specificity for “stemness” phenotype [168].
**CD106±** BM-MSCs [114], AD-MSCs [114], UCB-MSCs [123]	CD106, also known as vascular cell adhesion protein 1, is a cytokine-inducible cell surface protein capable of mediating adhesion and confers immunomodulatory properties to hBM-MSCs [169]. Migration and adhesion capacities conferred by CD106 to hMSCs happen through binding to hyaluronan (not specified tissue passage 14 cells) [170].
**CD106-** BM-MSCs [171], AD-MSCs [123], WJ-MSCs [124]	
**CD117-** BM-MSCs [114], UCB-MSCs [119]	CD117 is a receptor tyrosine kinase, also called tyrosine-protein kinase kit. CD117 ligand is stem cell factor, which upon binding to CD117 activates many signalling pathways to mediate cell proliferation [172]. SRC / Lyn pathway is affected by tyrosine-protein kinase kit, which allows for cell cycle progression in human hematopoietic cells [173]. Tyrosine-protein kinase kit also promotes human cardiac progenitor cell growth and migration through mitogen-activated protein kinase pathway [174].
**CD140b±** BM-MSCs [175], AD-MSCs [53]	CD140b is also known as platelet derived growth factor receptor β. CD140b knock-down using small interfering RNA and transfection in hAD-MSCs resulted in decreased cell proliferation, cell adhesion and migration, compared to CD140b+ hAD-MSCs. In co-culture with human retinal endothelial cells, CD140b- hAD-MSCs reduced the angiogenesis capacity of retinal endothelial cells, compared to their co-culture with CD140b+ hAD-MSCs [176].
**CD144-** BM-MSCs [123], AD-MSCs [109], UCB-MSCs [123]	CD144, also called vascular endothelial cadherin, is a cell-cell adhesion molecule expressed in human vascular endothelial cell junctions and responsible for vascular endothelium permeability [177]. It is involved in the clathrin-dependent endocytosis mechanism in human microvascular endothelial cells [178].
**CD146-** BM-MSCs [129], AD-MSCs [109], UCB-MSCs [129], UC-MSCs [129], G-MSCs [111]	CD146, also called melanoma cell adhesion molecule, is a highly glycosylated type I transmembrane protein and belongs to the immunoglobulin superfamily. It is mainly expressed in human malignant cancer cells [179] and at the junction of human endothelial cells for cell-cell cohesion [180].
**CD166±** BM-MSCs [181], AD-MSCs [109], UCB-MSCs [104], SF-MSCs [148], AM-MSCs [110], CH-MSCs [110], G-MSCs [111]	CD166, also called activated leukocyte cell adhesion molecule, is a type-I transmembrane protein belonging to the immunoglobulin receptor family. It is a marker used for hBM-MSCs [182], but its function on MSCs is unknown.
**CD166-** AM-MSCs [110], CH-MSCs [110], WJ-MSCs [124]	
**CD200±** BM-MSCs [183], UCB-MSCs, WJ-MSCs [184]	CD200 is a transmembrane surface glycoprotein that delivers immunoregulatory signals through binding to its receptor CD200R, present on human cells from the myeloid lineage. Binding of CD200R from human T-lymphocytes to CD200 from hMSCs (i.e. AD-MSCs and WJ-MSCs) induces immunosuppressive and anti-inflammatory effects [184]. CD200+ hBM-MSCs express high mRNA levels of osteogenic markers [185]. Additionally, an increase of CD200 protein expression was reported upon osteogenic or pro-inflammatory stimulation through nuclear factor-kappa B pathway [185]. Immunomodulation of hP-MSCs depends on the CD200 gene expression and was demonstrated in a stroke model [186].
**CD200-** BM-MSCs [53,183,187], AD-MSCs [53,187], UCB-MSCs [188]	
**CD248±** BM-MSCs, AD-MSCs [53]	CD248, also called endosialin, is a transmembrane receptor that binds to ECM components (i.e. fibronectin, collagen type I, collagen type IV) and has been shown to mediate cell adhesion and migration in Chinese ovary hamster cells [189]. In human and mouse hepatic cells, CD248 has been associated with cell proliferation following injury [190]. CD248 is expressed in human blood vessels during embryogenesis and tumorigenesis [191].
**CD271±** AM-MSCs (small proportion of cells) [110], CH-MSCs (small proportion of cells) [110]	CD271 belongs to the tumour necrosis factor receptor superfamily and is expressed primarily by adult hMSCs (i.e. BM-MSCs and AD-MSCs) [192]. CD271+ hBM-MSCs have shown upregulation of genes associated to ECM, cell adhesion, osteogenesis, chondrogenesis, adipogenesis and haematopoiesis, when compared to CD271- hBM-MSCs [193]. Down-regulated genes were associated to WNT and TGF-β signalling, and cytokine / chemokine signalling pathways [193]. Down-regulation of anti-inflammatory genes and up-regulation of angiogenesis related genes were observed in CD271+ hAD-MSCs compared to CD271- hAD-MSCs [194]. This marker has been reported to disappear upon cell culture in hAM-MSCs and CH-MSCs [110].
**CD271-** BM-MSCs [110,175], AD-MSCs [195], UCB-MSCs [192], UC-MSCs [196], WJ-MSCs [192]	
**CD273±** BM-MSCs, AD-MSCs, UC-MSCs [187]	CD273 is an immunomodulatory surface protein. hUC-MSCs proved to be less immunogenic and more potent immunosuppressors than hBM-MSC and the proposed reason is the higher gene and protein expressions of CD200, CD273 and CD274 [197].
**CD273-** BM-MSCs, AD-MSCs [53]	
**Stro1±** BM-MSCs [198]	Stro-1 antigen is located on plasma membrane and is an antibody against hMSCs, which binds to heat shock protein cognate 70 [199]. Stro-1 is expressed by human endothelial cells but is by human fibroblasts and smooth muscle cells [200]. Stro1+ hBM-MSCs have shown superior inhibition of human T lymphocyte proliferation compared to unsorted hBM-MSCs [201]. Conditioned medium from Stro1+ hBM-MSCs applied to human cardiac muscle cells resulted in higher cardiac muscle cell proliferation and angiogenic properties compared to conditioned medium from unsorted hBM-MSCs [202].
**Stro1-** BM-MSCs [203], AD-MSCs [114], UC-MSCs [204]	
**HLA-ABC±** BM-MSCs [104], AD-MSCs [105], UCB-MSCs [104], UC-MSCs [129], G-MSCs [111]	HLA is a class I major histocompatibility antigen. HLA-ABC confers to hBM-MSCs the function of antigen presenting cells, when are exposed to viral peptides (i.e. Influenza A, Hepatitis C) [205]. HLA-ABC protein expression in hBM-MSCs decreases upon adipogenic differentiation and remains upon chondrogenic and osteogenic differentiations remained HLA-ABC positive [206].
**HLA-DR-** BM-MSCs [206], AD-MSCs [105], UCB-MSCs [123], WJ-MSCs [207], G-MSCs [111]	HLA-DR is a class II major histocompatibility antigen. Those antigens are expressed by hBM-MSCs upon inflammation stimuli (i.e. IFN-α, IFN-γ, IL-1β, TNF-α), but HLA-DR protein expression does not increase in hAD-MSCs and hWJ-MSCs upon this stimulation [207]. HLA-DR protein expression on hBM-MSCs did not have negative impact in clinical studies [208].
**CSPG4±** UCB-MSCs [192], UC-MSCs [129], WJ-MSCs [192]	CSPG4 is a cell surface proteoglycan. In tumour cells, CSPG4 plays a role in cell proliferation [209] and adhesion [210] and promotes tumour motility [211]. Also it supports angiogenesis by promoting human endothelial cell migration and morphogenesis [212].
**CSPG4-** BM-MSCs [129], UCB-MSCs [129]	
**ABCG2-** BM-MSCs [114], AD-MSCs [105]	Member of the ATP binding cassette transporters, which play a role in promoting human hematopoietic stem cell proliferation and the maintenance of the immature human hematopoietic stem cell phenotype [213], along with being a regulatory protein of early human hematopoietic development [214].
**MSCA-1±** BM-MSCs [215], WJ-MSCs [216]	MSCA-1, also known as tissue non-specific alkaline phosphatase, expression on hBM-MSCs increases the number of colony forming units, compared to unsorted hBM-MSCs [217]. TNF-α and IL-β1 increase TNAP activity in hBM-MSCs, which in turn increases the mineralisation capacity of these cells [218]. Other studies have also associated TNAP activity to higher osteogenic differentiation of hBM-MSCs [219].
**SSEA-4-** BM-MSCs [136], AD-MSCs [136], UCB-MSCs [196], G-MSCs [111]	SSEA-4 is a glycolipid antigen that is used for human embryonic / pluripotent stem cell identification [220,221]. SSEA-4 protein expression has been associated with the loss of cell-cell interactions and the gain of a migratory phenotype in human solid cancer cell lines [222].

Abbreviations: ABCG: ATP-binding cassette super-family G member 2, AD-MSCs: adipose derived mesenchymal stromal cells, AM-MSCs: amnion derived mesenchymal stromal cells, ATP: adenosine triphosphate, BM-MSCs: bone marrow mesenchymal stromal cells, CD: cluster of differentiation, CH-MSCs: chorion mesenchymal stromal cells, CSPG4: chondroitin sulphate proteoglycan 4, ECM: extracellular matrix, G-MSCs: gingival mesenchymal stromal cells, h: human, HLA: human leukocyte antigen, IFN: interferon, IL: interleukin, mRNA: messenger ribonucleic acid, MSCs: mesenchymal stromal cells, MSCA: mesenchymal stem cell antigen, P-MSCs: placenta mesenchymal stromal cells, RNA: ribonucleic acid, SF-MSCs: synovial fluid mesenchymal stromal cells, SSEA: Stage-specific embryonic antigen, TGF: transforming growth factor, TNF: tumor necrosis factor, TNAP: tissue non-specific alkaline phosphatase, UC-MSCs: umbilical cord mesenchymal stromal cells, UCB-MSCs: umbilical cord blood mesenchymal stromal cells, WJ-MSCs: Wharton's Jelly MSCs.

With respect to TCs, the situation is far more complex. Firstly, one should note that to-date, no TC-specific marker has been identified. Secondly, the two widely used TC extraction protocols (i.e. collagenase digestion [57] and migration [58]) not only can differentially affect marker expression [59], but also do not ensure purity, considering that tendon tissues also home tendon stem / progenitor cells [60] and synovial cells [61]. In an attempt to standardise TC characterisation, it was suggested that along the traditional characteristics (e.g. plastic adherent elongated cells) and markers (e.g. collagen type I, collagen type III, collagen type V, decorin, scleraxis, tenomodulin, tenascin-C, thrombospondin 4, mohawk), trans-differentiation markers (e.g. chondrogenic markers, such as collagen type II, aggrecan, SOX9, COMP; osteogenic markers, such as osteocalcin, osteopontin, RUNX2, alkaline phosphatase; adipogenic markers, such as PPARγ; and myofibroblast markers, such as α-SMA) should also be assessed [47]. Table 3 provides a list of indicative human TC markers, along with their function and method used to assess them.

**Table 3:** Indicative examples of hTC markers, methods used to assess them and function (in tendon and/or other tissues).

Marker and method used to assess them	Function
**Collagen I** Immunofluorescence [223] Western blot [50] ELISA [224] Gene expression [50,59] Transcriptomics [225]	Collagen I is an ECM protein that belongs to the fibril-forming collagen family and represents 65–80 % of the dry mass in human supraspinatus and biceps flexor tendons [226]. Collagen type I confers the mechanical properties of the human supraspinatus tendon, including resistance to high tensile and compression loads [227]. The property of human palmaris and plantaris tendons to spread is directly related to their straight and parallel collagen organisation [228].
**Collagen III** Immunofluorescence [223] Western blot [50] Gene expression [50,59] Transcriptomics [225]	Collagen III is an ECM protein that belongs to the fibril-forming collagen family. Collagen type III fibrils are thinner than collagen I and co-interact with collagen type I during fibrillogenesis to regulate fibril formation and growth [229]. Collagen type III represents around 3 % of the total collagen in human supraspinatus tendon [226]. Tendinitis in human supraspinatus tendon increases collagen type III content [226].
**Collagen V** Immunofluorescence [223] Gene expression [230] Transcriptomics [225]	Collagen type V is an ECM protein that belongs to the fibril-forming collagen family. Collagen type V influences the nucleation and number and diameter of collagen type I fibrils in mouse flexor digitorum longus [231]. Ehlers-Danlos syndrome is associated with collagen type V mutation [232].
**Decorin** Western blot [50] Gene expression [50,59,230] Transcriptomics [225]	Decorin is a small chondroitin-dermatan sulphate proteoglycan. Decorin regulates fibril development, growth, fusion and orientation in tendon development. In aging tendons, the continued expression of decorin results in the presence of a subpopulation of larger in diameter fibrils that may be more susceptible to damage and reduced alignment and mechanical properties [233].
**Tenomodulin** Immunofluorescence [85,234] Gene expression [50]	Tenomodulin is a type II transmembrane protein that has been shown in vivo in a tenomodulin knockout mouse model to be necessary for tenocyte proliferation and tendon maturation (Achilles and patellar tendons) and therefore it is considered as a late tendon marker [235]. Tenomodulin role during tendon healing was analysed in an Achilles tendon tenomodulin knockout mouse model, where a role in adipocyte accumulation, neovascularisation and increased cell apoptosis were reported [236]. Tenomodulin is involved in crosslinking and biomechanical properties of collagen type I, as described in an Achilles tendon tenomodulin knockout mouse model [234]. Three tenomodulin isoforms have been identified in human tendon samples (i.e. flexor carpi radialis, biceps brachii and flexor digitorum profundus tendons) and knock-down (through small interfering RNA) of tenomodulin in hTCs has been shown to result in decreased hTC proliferation [237]. Tenomodulin protein expression in human Achilles tendon stem cells is lost in two-dimensions culture, but its expression can be restored by mechanical stretching [234].
**Scleraxis** Western blot [76] Gene expression [50,59,230] Transcriptomics [225]	Scleraxis is a transcription factor necessary for normal development of force-transmitted tendons in mouse [238]. Scleraxis knockout in mice resulted in small and disorganised flexor digitorium profundus tendons [238]. Scleraxis expression is maintained by TGF-β / Smad2/3 signalling pathway [239]. Mechanical stretch induces TGF-β release from the ECM [239]. During a temporary or gradual loss of tensile load, scleraxis expression is lost in mouse Achilles tendon [239,240]. Transduction of scleraxis gene into a hBM-MSC line triggered their commitment towards tenogenic phenotype [241].
**Tenascin C** Immunofluorescence [242] Western blot [50] Gene expression [50,59,230] Transcriptomics [225]	Tenascin C is a glycoprotein localised at the myotendinous and fascicle tendon areas of chick embryo wing bud [243] and around organised, fibrous regions of human supraspinatus tendon [244]. Tenascin C has elastic properties [245]. Bovine deep flexor tendon was used to demonstrate anti-adhesion and adaptation of compressive loads properties of tenascin C [246]. Healthy human supraspinatus tendon expressed a 200 kDa tenascin C (whilst their degenerated counterparts expressed a 300 kDa tenascin C) and it was shown to induce TC proliferation and fibrocartilage formation [244].
**Mohawk** Gene expression [247,248] Immunohistochemistry [249] Immunocytochemistry [249]	Mohawk is a transcription factor that regulates collagen type I protein production in Achilles and tail tendons of mohawk knockout mouse [250]. Tail and limb tendons from mohawk knockout mouse exhibited abnormal tendon sheaths and expressed significantly reduced amounts of mRNA levels of collagen type I, fibromodulin and tenomodulin, compared to control mice [251]. Transduction of hBM-MSCs to insert mohawk gene demonstrated to increase tenogenesis of those cells [252]. Low mRNA levels of mohawk were associated with tendinopathy in human [253].
**Biglycan** Gene expression [230] Transcriptomics [225]	Biglycan is a small leucine-rich repeat proteoglycan. Biglycan has been linked to many aspects of mouse patellar tendon biomechanical and structural properties, including insertion modulus, maximum stress, dynamic modulus, stress relaxation and increased collagen fibre alignment during loading in a biglycan knockout mouse model, where all those functions were impaired [254].
**Fibronectin** ELISA [224] Gene expression [59,234] Transcriptomics [225]	Fibronectin is a glycoprotein that allows for cellular adhesion and spreading in rat epitenon fibroblasts from flexor tendon [255]. In human tendon fibroblasts, fibronectin colocalizes with collagen type I, collagen type VI and collagen type XII [256].
**Thrombospondin-4** Western blot [257] Gene expression [257] Transcriptomics [225]	Thrombospondin-4 is a glycoprotein that has been shown to form complexes with cartilage oligomeric matrix protein in equine superficial digital flexor tendon [258]. Thrombospondin-4 has been shown to be specific to human tendon tissue, along with tenomodulin [259].

Abbreviations: ECM: extracellular matrix, ELISA: enzyme linked immunosorbed assay, hBM-MSCs: human bone marrow mesenchymal stromal cells, mRNA: messenger ribonucleic acid, RNA: ribonucleic acid, Smad: mothers against decapentaplegic, TC: tenocytes, TGF: transforming growth factor.

## 4 MSC / TC co-cultures

Considering the number of variables among the different publications (e.g. species; tissue; media; cell type density, ratio, extraction method, passage; time points; read outs; etc.), a direct comparison between different papers is not possible. To that effect, Table 4 summarises results of individual studies and here, we discuss some trends, but also contradictions observed in the literature. With respect to cell ratio, in general, the 1 to 1 MSCs to TCs ratio has been shown to significantly increase cell proliferation, protein synthesis and expression of tenogenic markers [62,63]. It is unfortunate that a higher MSCs to TCs ratio did not demonstrate significant improvement, considering the low number of functional TCs available. With respect to direct versus indirect cultures, studies advocate the use of direct cultures [62,64], due to the direct cell-to-cell communication enabled by gap junctions [65,66]. This theory finds support through analogies drawn from studies involving co-cultures of MSCs with chondrocytes or osteoblasts, where such interactions induced chondrogenic [67] or osteogenic [68], respectively, MSC differentiation. The second reason is based on the well-established in the literature paracrine effect of MSCs in tissue repair and regeneration [[69], [70], [71], [72]]. In tendon context, as both bone marrow (BM) and adipose derived (AD) -MSCs have been shown to secrete trophic factors and stimulatory molecules inducive to tendon extracellular matrix production, remodelling and healing, their use in direct cultures with TCs has been advocated as a means to exploit the activation of paracrine signalling cascades for effective TC phenotype maintenance and/or tenogenic induction [[73], [74], [75]].

**Table 4:** Direct, indirect and CM MSCs with TC co-culture systems.

Experimental Design	Comments
**Direct, human cells** AD-MSCs (passage 2 to 4) TCs (passage 2 to 3, tissue not mentioned) Ratio AD-MSCs to TCs: 1 to 1 Cell number: 2000 cells/cm^2^	Up-regulation in mRNA levels of COLI, TNC and MMP-3 in co-culture in comparison to TCs alone at day 7. No effect of co-culture on mRNA levels of SCX, MKX, DCN, MMP-1, MMP-2 and TIMP-1 compared to TCs alone at any time point. Co-culture increased protein expression of collagen type I at day 7 and day 14 compared to TCs alone and decreased collagen type III at day 7 compared to AD-MSCs alone. A reduction of secreted MMP-3 at day 3 and day 14 was observed compared to AD-MSCs alone and TCs alone at the corresponding time point. Co-culture led to reduced aspect ratio compared to both AD-MSCs alone and TCs alone. Co-culture also shown a reduction in proliferation compared to AD-MSCs alone at day 7 and day 14 [247].
**Direct, human cells** AD-MSCs (less than passage 6) Flexor tendon TCs (passage 3 to 5) Ratio AD-MSCs to TCs: 1 to 1, 1 to 3, 3 to 1 Cell number: 5000 cells/cm^2^ **Indirect, human cells** AD-MSCs (less than passage 6) Flexor tendon TCs (passage 3 to 5) cultured in the insert Ratio AD-MSCs to TCs: 1 to 1 Cell number: 5000 cells/cm^2^ **CM, human cells** CM was harvested from TCs (passage 3 to 5, cell number 5000 cells/cm^2^) cultured from 70 % to 90 % confluency for 24 h and CM was applied on AD-MSCs alone (less than passage 6, cell number 5000 cells/cm^2^) Ratio fresh medium to CM: 1 to 1	Increased proliferation in direct co-culture ratio 3 to 1 at day 6 and day 7 and in co-culture ratio 1 to 3 at day 7, compared to AD-MSCs alone and TCs alone. Direct co-culture up-regulated mRNA levels of SCX (ratio 1 to 3) and TNC (all ratios) compared to AD-MSCs alone. Following this analysis, directly co-cultured AD-MSCs were separated from TCs by flow analysis cell sorting. Sorted AD-MSCs demonstrated an up-regulation of mRNA levels of SCX and COLIII (ratios 1 to 1 and 3 to 1) and TNC (all ratios), compared to AD-MSCs alone. TC derived CM increased mRNA levels of COLIII and TNC, compared to AD-MSCs alone in basal medium. Indirect co-culture increased mRNA level of TNC and COLIII, compared to AD-MSCs alone [64].
**Indirect, human cells** BM-MSCs (passage 2 to 5) Hamstring tendon TCs (passage 2 to 5) Ratio BM-MSCs to TCs: 1 to 1 Cell number: 25,000 TCs in the well and 25,000 BM-MSCs in the insert and 25,000 TCs in the insert and 25,000 BM-MSCs in the well **CM, human cells** CM was harvested from BM-MSCs alone and TCs alone cultures (25,000 cells, passage 2 to 5) after 3 days of culture Ratio fresh medium to CM: not specified TCs were exposed for 24 h to CM from TCs or CM from BM-MSCs.	An increase in mRNA levels in co-cultured TCs was observed for MMP-1 (donor 1, day 7 and day 14, donor 3 at day 14, donor 4 at day 7), MMP-3 (donor 1 and donor 3 at day 7), MMP-13 (donor 1 at day 7 and day 14, donor 3 at day 14, donor 4 at day 7), ADAMTS-4 (donor 1 at day 7, donor 3 at day 7 and day 14) compared to TCs alone. Co-cultured BM-MSCs showed an up-regulation of TNMD (donor 2 and donor 4 at day 7 and day 14), ACAN (donor 2 at day 7, donor 4 at day 7 and day 14) and ADAMTS-5 (donor 2, donor 3, donor 4 at day 7) compared to BM-MSCs alone. Collagen / non collagen ECM protein ratio was enhanced in co-cultured TCs compared to TCs alone (donor 3 at day 7, donor 4 at day 7 and day 14). Using CM from either BM-MSCs or TCs, TC aspect ratio was augmented compared to basal medium. CM from BM-MSCs contained more TGF-β1 than CM from TCs [260].
**Indirect, human cells** BM-MSCs (passage 3) Tendon explants from degenerative rotator cuff Ratio: 4000 BM-MSCs/cm^2^ in the well and 0.005, 0.01, 0.025, 0.05, 0.1, 0.25, 0.5, 1.0, 2.0 g of explant in the insert	mRNA levels of COLI (0.1 g), COLIII (0.05, 0.1, 0.25, 0.5 g), SCX (0.5 g), TNMD (0.05, 0.1, 0.25, 0.5 g), DCN (0.1, 0.25, 0.5 g) and TNC (0.5 g) were up-regulated in co-culture compared to BM-MSCs alone. mRNA levels of COLI (1.0, 2.0 g), SCX (2.0 g), DCN (2.0 g), ACAN (0.025, 0.05, 0.1, 0.25, 0.5, 1.0, 2.0 g) in co-culture were down-regulated compared to BM-MSCs alone. No effect of co-culture on mRNA level of ALP was reported compared to BM-MSCs alone. No effect on cell viability was observed in co-culture compared to BM-MSCs alone and cellular senescence was enhanced in some tendon-explant cultures (0.05, 0.1, 0.2 g) compared to BM-MSCs alone [261].
**Indirect, human cells** AD-MSCs (passage 2 to 3) Tendon explants from knee sartorius or gracilis tendon. Ratio: 10,000 CE-MSCs/well and 25 mm^3^ of explant in the insert	At day 7, a down-regulation in protein expression of collagen type III and tenascin C was observed in co-culture compared to AD-MSCs alone. Collagen type III staining was higher at day 14 of co-culture compared to day 7 of co-culture. Collagenase activity was enhanced in co-culture compared to AD-MSCs alone and tendon explant alone. Protease activity was increased in co-culture compared to AD-MSCs alone. A reduction in nucleus aspect ratio in co-culture was observed compared to AD-MSCs alone [262].
**Indirect, equine cells** AD-MSCs (passage 2) or SVF Flexor digitorum superficialis tendon TCs (passage 2 to 6) Ratio: 0, 1000, 2000, 3000, 4000 CE-MSCs in the insert and 50,000 TCs in the well 0, 100,000, 250,000, 500,000 SVF in the insert and 10,000 TCs in the well	Co-cultured TCs with AD-MSCs demonstrated an up-regulation in mRNA of COLI and COLIII at all ratios and an increase in mRNA of COMP and DCN when 1000 and 4000 CE-MSCs were use, compared to TCs alone. Co-cultured TCs with SVF showed an up-regulation in mRNA of COLIII (all ratios) and a down-regulation in COMP (all ratios), compared to TCs alone [263].
**Indirect, equine cells** BM-MSCs (passage 3) Superficial digital flexor tendon explants Ratio: 1000 BM-MSCs/cm^2^ in the well and 2–3 mm^3^ explant in the insert	Upregulation in mRNA levels of COLI, DCN, TNC, TNMD was observed in co-culture compared to BM-MSCs alone [264].
**CM, equine cells** CM was harvested from 22,857 BM-MSCs/cm^2^ (passage 2) after 2 days in culture without serum and antibiotics. The CM was applied to 400,000 superficial digital flexor tendon TCs (unknown passage)/well Groups: healthy control group, inflamed untreated group (inflammation medium), CM group, extracellular vesicle group, protein fraction group. Ratio fresh medium to CM: 1 to 2	TCs were cultured for 24 h either with basal medium (i.e. healthy control) or with inflammation medium (i.e. serum-free medium containing 10 ng/ml TNF-α and 10 ng/ml IL-1β). CM group showed the highest differential gene expression (120 genes differentially expressed, 42 genes down-regulated, 78 genes up-regulated) on inflamed TCs versus extracellular vesicle group (33 genes differentially expressed, 16 genes down-regulated, 17 genes up-regulated) and protein fraction group (57 genes differentially expressed, 17 genes down-regulated, 40 genes up-regulated) compared to the untreated inflamed group [265].
**Indirect, equine cells** AD-MSCs (passage 3) Superficial digital flexor tendon peritenon or tendon TCs (passage 3) Ratio: 5263 CE-MSCs/cm^2^ in the insert and 5263 TCs/cm^2^ in the well	No variations in mRNA levels of tenogenic (SCX, MHK, BGN, DCN, COLI, FMOD), ECM (i.e. LOX), and cell proliferation (Ki67) related markers were observed by co-culturing either AD-MSCs / tendon TCs and AD-MSCs / peritenon TCs compared to AD-MSCs alone. Significant decrease in mRNA level of COLI and significant increase in mRNA levels of BGN was observed at day 5 of co-culture compared to day 2 co-culture (for both TCs) [266].
**Direct, rat cells** BM-MSCs (passage was not specified) Achilles tendon TCs (passage was not specified) Ratio BM-MSCs to TCs: 1 to 1, 1 to 3, 2 to 3 (Total cell number: 20,000 cells/well) **Indirect, rat cells** 20,000 BM-MSCs in the insert and 20,000 TCs in the well	Direct co-culture (ratio 1 to 1) decreased mRNA levels of SCX and increased RUNX2 and SOX9, without variation of PPARγ, compared to BM-MSCs alone and TCs alone. The ratio 1 to 1 in direct co-culture enhanced cell proliferation compared to BM-MSCs alone and TCs alone. Indirect co-culture did not show differences in mRNA levels of SCX, RUNX2, SOX9 and PPARγ compared to BM-MSCs alone and TCs alone [267].
**Indirect, rat cells** BM-MSCs (passage 3) Achilles tendon TCs (passage 3) Ratio: 20,000 BM-MSCs in the well and 20,000 TCs in the insert	Co-culture led to an up-regulation in mRNA of COLI at day 14 and day 21, COLIII at day 14 and day 21, TNC at day 7, day 14 and day 21, SCX at day 7, day 14 and day 21 compared to BM-MSCs alone. An increase in co-cultured BM-MSCs proliferation was observed compared to BM-MSCs alone [268].
**Indirect, rat cells** BM-MSCs (passage 2) Achilles tendon TCs (passage 4 or 7) Ratio: 500,000 BM-MSCs or TCs (control group) in the well and 10,000 TCs in the insert **CM, rat cells** The CM was harvested from the indirect co-culture described above and from BMSCs alone (500,000 cells) and was applied to TCs. Only CM was used, no mix with fresh medium.	Indirect co-culture demonstrated an increased proliferation over time compared to TCs alone. Co-culture demonstrated faster migration from the wound edges created by tip scrapping compared to TCs alone. CM from co-culture on TCs alone demonstrated enhanced proliferation in this group compared to CM from BM-MSCs alone and CM from TCs alone groups. CM from co-culture showed higher pre-albumin protein level compared to CM from BMSCs alone. CM from BMSCs alone group showed higher plasminogen and hear-keratin compared to CM from co-culture group [269].
**Indirect, mouse cells** AD-MSCs (passage 2 to 4) Tail tendon TCs (passage 2 to 4) Ratio: 12,000 CE-MSCs in the well and 26,000 TCs in the insert M0, M1, M2 macrophages (no further information was provided)	TCs / M1 macrophages co-culture increased drastically mRNA levels of matrix degradation markers (MMP-1a, MMP-3, MMP-13), inflammation (TNF-α, IL-1β, COX2) and up-regulation of proteins associated to pro-inflammation (PGE2, IL-β1, TNF-α), along with a down-regulation of tendon related genes (COLI, COLIII, BGN, DCN, TNMD) were observed compared to TCs alone. AD-MSCs / TCs / M1 macrophages tri-culture at day 1 did not show important mRNA differences compared to AD-MSCs / macrophages M1 co-culture. At day 5, mRNA decrease of COX2 and SCX and an up-regulation of COLI in AD-MSCs / TCs / M1 macrophages tri-culture compared to AD-MSCs / M1 macrophages co-culture were observed. AD-MSCs / TCs / M0 macrophages tri-culture at day 1 showed mRNA up-regulation of COLI and mRNA down-regulation of MMP-1a, MMP-3, SCX and TNMD compared to AD-MSCs / M0 macrophage co-culture. 5 days of culture did not considerably affect mRNA levels in AD-MSCs / TCs / M0 macrophage tri-culture compared to AD-MSCs / M0 macrophage co-culture. When AD-MSCs were added to TCs treated with IL-1β, AD-MSCs failed to protect TCs at all time points studied compared to co-culture AD-MSCs / TCs not treated with IL-1β (i.e. no significant changes in mRNA of TNF-α, IL-1β, MMP-1, MMP-3, and protein expression of IL-1β). Yet, on protein level, AD-MSCs did reduce TNF-α and IL-β1 in AD-MSCs / TCs / M1 macrophage tri-culture after 5 days compared to TCs / M1 macrophage co-culture. AD-MSCs induced a phenotypic drift from M0 to M2 macrophages at day 1 and day 5 of AD-MSCs / TCs / M0 macrophages tri-culture compared to TCs / M0 macrophages co-culture as observed by the significant decrease of CD301 protein in AD-MSCs / TCs / macrophages tri-culture compared to TCs / macrophages co-culture [270].
**Direct, canine cells** AD-MSCs (passage was not specified) TCs (passage and tissue were not specified) Total cell number: 1 × 10^6^ cells Ratio of AD-MSCs to TCs: 9 to 1, 7 to 3, 1 to 1 **CM, canine cells** The CM from TCs or medium with IGF-1 and TGF-β1 was applied on MSCs at 1 × 10^6^ cells	Direct co-culture showed an up-regulated protein expression of collagen type I, collagen type III, tenomodulin, integrin β1, MAPK-signalling-pathway-associated-proteins Shc and p-ERK 1/2 compared to AD-MSCs alone at days 7 and 14 for all ratios. AD-MSCs cultured with IGF-1 and TGF-β1, CM on AD-MSCs and direct co-culture (ratio 1 to 1) all resulted in similar protein levels increase of collagen type I, tenomodulin, scleraxis, integrin β1, Shc and p-ERK 1/2 compared to AD-MSCs alone in normal medium at day 7 and day 14 [62].

Abbreviations: ACAN: Aggrecan, ADAM: a disintegrin and metalloproteinase, AD-MSCs: adipose derived mesenchymal stromal cells, ALP: alkaline phosphatase, BGN, biglycan, BM-MSCs: bone marrow stem cells, CD: cluster of differentiation, COL: collagen, COMP: cartilage oligomeric matrix protein, COX: Cyclooxygenase, CM: conditioned medium, DCN: decorin, ECM: extracellular matrix, ERK: extracellular signal-regulated kinase, FMOD: fibromodulin, IGF: insulin-like growth factor, IL: interleukin, Ki-67: marker of proliferation Kiel 67, LOX: lysyl oxidase, MAPK: mitogen-activated protein kinase, MHK: mohawk, MKX: Homeobox protein Mohawk, MMP: matrix metalloproteinase, PGE2: prostaglandin E2, PPAR: peroxisome proliferator-activated receptors, RNA: ribonucleic acid, RUNX2: runt-related transcription factor 2, SCX: scleraxis, Shc: Src homology and collagen, TCs: tendon cells, SOX: SRY-Box transcription factor 9, TGF: transforming growth factor, TIMP: tissue inhibitor of metalloproteinase, TNC: tenascin, TNF: tumor necrosis factor, TNMD: tenomodulin, SVF: stromal vascular fraction.

As we are entering the era of multifactorial tissue engineering, more elegant and bioinspired in nature approaches are slowly emerging to drive MSCs towards tenogenic lineage in a co-culture setting (Table 5). Considering that TCs naturally reside in a low oxygen environment, which has been shown to prolong the in vitro function of adult TCs [76], foetal progenitor TCs [77] and tendon-derived stem cells [78], it is not surprising that cellular normoxia has shown promise when was probed under co-culture conditions. Indeed, when human AD-MSCs were indirectly co-cultured with rat Achilles tendon TCs (1 to 1 ratio; MSCs on the plate and TCs on the membrane of the transwell chamber) under 20 % and 2 % oxygen tension, cultures at 2 % oxygen induced significant increase in cell proliferation and gene expression levels of COLI, COLIII, TNMD, THBS4, SCX and HIF‐1α [79]. Obviously, it is unclear whether the same outcome will be achieved if human AD-MSCs are to be cultured with human Achilles tendon TCs. Considering that tendon tissues are subjected to mechanical stimulation [80,81], co-culture of rabbit BM-MSCs from the proximal tibia with semitendinosus tendon TCs (1 to 1 ratio) on collagen type I coated substrates under 10 % strain for 48 hours at frequency of 10 cycles / minute with each cycle consisting of 2 seconds of stretch and 2 seconds of relaxation resulted in increased cell proliferation and upregulation of COLI, COLIII, ALP, OPN, TNC and TNMD and unaffected COLII gene expression [82]. One cannot but note that upregulation of bone specific markers (i.e. ALP [83] and OPN [84]) may indicate incomplete differentiation. In a far more complex study, anisotropic electrospun scaffolds were loaded with human AD-MSCs, human TCs from the long head of biceps tendons and human umbilical vein endothelial cells (ratio of 2 to 2 to 1, respectively) and subjected to cyclic uniaxial strain (in the direction of the aligned scaffold) with 4 % elongation and 0.5 Hertz frequency for 2 hours per day for 12 days. Gene expression analysis revealed that this tri-culture / scaffold system induced the highest SCX, TNC and TNMD gene expression compared to human AD-MSCs alone group, human AD-MSCs with HUVECs co-culture group and human AD-MSCs with TC co-culture group. The co- and the tri- culture / scaffold / mechanical stimulation system significantly increased total collagen content and upregulated the gene expression of SCX, TNC, COLI, COLIII, TNMD and VEGFA compared to static culture [85]. It is unfortunate the trans-differentiation markers were not assessed. Taken together, these data strongly advocate the use of appropriate culture conditions to effectively induce tenogenic induction, as has been suggested previously [86,87].

**Table 5:** Multifactorial TC co-culture systems.

Experimental Design	Comments
**Direct, human cells** AD-MSCs (passage 4 to 6) primed with TGF-β3 for tenogenic differentiation Bicep tendon TCs (passage 4 to 6) HUVECs (passage 4 to 6) Ratio AD-MSCs to TCs: 1 to 1 Ratio AD-MSCs to HUVECs: 4 to 1 Ratio AD-MSCs to TCs to HUVECs: 2 to 2 to 1 PCL/PLA electrospun scaffold Cyclic uniaxial strain with 4 % elongation along the direction of scaffold fibres, 0.5 Hz frequency for 2 h per day for 12 days on the tri-culture	AD-MSCs / TCs co-culture led to the up-regulation of mRNA levels of SCX, TNC and TNMD and decreased mRNA levels of COLIII compared to AD-MSC. AD-MSCs / HUVECs co-culture showed an augmentation of mRNA levels of SCX, COLIII, TNMD, VEGFA and ANGPT2 compared to AD-MSC. AD-MSCs / TCs / HUVECs tri-culture increased mRNA levels of SCX, TNC, COLIII, TNMD, VEGFA and ANGPT2 compared to AD-MSC. All co-cultures and tri-cultures showed less mRNA levels of COLI compared to AD-MSC scaffold. In mechanically stimulated tri-culture, up-regulation in mRNA levels of COLI, COLIII, SCX, TNC, TNMD and VEGFA were observed (no effect on ANGPT2) along with higher collagen content and protein levels of tenomodulin and collagen type I compared to tri-culture in static condition [85].
**Direct, human cells** AD-MSCs (passage 2 to 5) osteogenically differentiated Unspecified tissue TCs (passage 2 to 5) Ratio AD-MSCs to TCs: 1 to 1, with 2000,000 cells per scaffold Magnetic responsive hydrogel composed of methacrylated chondroitin sulphate, platelet lysate and magnetic nanoparticles containing cells. EMF, oscillating magnet array system of 2 Hz frequency and 0.2 mm displacement	One scaffold was fabricated with TCs and another scaffold was made with osteogenically differentiated AD-MSCs to create a tendon-to-bone interface. The two scaffolds were attached to each other, allowing for direct contact and creation of an interface. EMF increased mRNA levels of DCN from day 7 to day 21 in the tendon scaffold compared to tendon scaffold not exposed to EMF. EMF decreased protein levels of collagen type I and tenascin C in tendon, interface and bone scaffolds compared to scaffold not exposed with EMF for the respective scaffold. EMF up-regulated protein level of osteopontin in tendon scaffold and down-regulated its expression in the bone scaffold compared to scaffold not exposed to EMF for the respective scaffold. Tendon, interface and bone scaffolds not exposed to EMF were similar to each other in terms of mRNA levels of COLI, DCN and OPN expression across all time points. The interface presented all genes (COLI, DCN, OPN) and proteins expressions (tenascin C, osteopontin) [242].
**Direct, human cells** BM-MSCs (passage 4) Unspecified tissue TCs (passage 4) Ratio BM-MSCs to TCs: 1 to 1, with 5000 cells/cm^2^ PRP (10 % v/v, activated with 10 % calcium chloride	Co-culture with PRP up-regulated protein level of fibronectin and aggrecan compared to co-culture without PRP and compared to TCs alone. Collagen type I was increased in co-culture without PRP compared to TCs alone without PRP. Co-culture with PRP enhanced further this augmentation. PRP improved cell migration in co-culture compared to TCs alone and co-culture without PRP at 4 h and 8 h. At 24 h, all conditions stopped showing cell migration. Co-culture with PRP enhanced cell repopulation of the scratched area compared to co-culture without PRP and TCs alone at 4 h, 8 h and 24 h [224].
**Direct, human cells** AD-MSCs (passage 2 to 3) osteogenically differentiated Unspecified tissue TCs (passage 2 to 4) Ratio AD-MSCs to TCs: 1 to 1, with 2000 cells/cm^2^ Ratio osteogenic medium to basal medium: 1 to 1, 1 to 0	Comparing TCs alone and pre differentiated AD-MSCs alone, both expressed the same mRNA markers. Co-culture with osteogenic medium / basal medium mix led to higher mRNA levels of RUNX2 at day 7 compared to pre-osteoblast AD-MSCs alone, without affecting TNAP. Co-culture with osteogenic medium decreased mRNA levels of TNC compared to TCs alone at day 14. mRNA level of ACAN was increased at day 7 in co-culture with osteogenic medium compared to pre-osteoblast AD-MSCs alone and TCs alone in osteogenic medium. Co-culture exposed either to osteogenic medium or osteogenic medium / basal medium mix up-regulated mRNA level of COMP compared to pre-osteoblast AD-MSCs alone and TCs alone in those media at day 14. At day 14, mRNA level of COLI of co-culture with osteogenic medium was higher than pre-osteoblast AD-MSCs alone and TCs alone with osteogenic medium. At day 7, co-culture with osteogenic medium or osteogenic medium / basal medium mix shown lower mRNA level of COLI compared to TCs alone with those media. mRNA level of COLIII was reduced in co-culture (osteogenic medium and osteogenic medium / basal medium mix) compared to TCs alone at day 7 but the opposite was observed at day 14. At the protein level, aggrecan was absent from pre-osteoblast AD-MSCs alone and TCs alone compared to the high expression found in co-culture at day 7 and 14 (independently of the medium used). Pre-osteoblast AD-MSCs alone did not express collagen type II at day 7 compared to co-culture, which stained strongly for it at both time points (independently of the medium used). Osteocalcin was stained by pre-osteoblast AD-MSCs alone and co-culture at day 7 but expression was lost for pre-osteoblast AD-MSCs alone at day 14, while co-culture was unaffected (independently of the medium used). Osteogenic medium induced the loss of tenascin C expression in co-culture at day 14 compared to BM, while tenascin C expression remained in TCs alone. At day 7, mineralisation was similar between co-culture and pre-osteoblast AD-MSCs but drastically higher than TCs (independently of the medium used) [271].
**Indirect, human cells** MB-MSCs (passage was not specified) Achilles tendon TCs (passage was not specified) Ratio MB-MSCs to TCs: 10,000 MB-MSCs in the well and 10,000 TCs in the insert Oxygen tension: 2 % and 20 %	Co-culture at 20 % oxygen tension increased mRNA levels of COLI, COLIII, TNC, SCX, THBS4 at day 14 and day 21 compared to MB-MSCs alone at 20 % oxygen tension. Co-culture combined with at 2 % oxygen tension resulted in no mRNA level difference compared to co-culture at 20 % oxygen tension. Co-culture at 20 % oxygen tension enhanced protein levels of collagen type I and thrombospondin 4 compared to MB-MSCs alone at 20 % oxygen tension and at 2 % oxygen tension up-regulated further those protein expressions compared to 20 % oxygen tension co-culture condition. Co-culture improved proteoglycan content compared to MB-MSCs alone at 20 % oxygen tension and 2 % oxygen tension increased further proteoglycan content compared to MB-MSCs alone at 2 % oxygen tension [272].
**Indirect, human and rat cells** hAD-MSCs (passage 3 to 6) Achilles tendon rat TCs (passage 3 to 5) Ratio hAD-MSCs to rat TCs: 10,000 hAD-MSCs in the well and 10,000 rat TCs in the insert Oxygen tension: 2 % and 20 %	Co-culture led to up-regulation of mRNA levels of COLI, COLIII, SCX and THBS4 at day 14 and day 21 at 2 % oxygen tension compared to co-culture at 20 % oxygen tension. Increased mRNA level of TNMD was observed in co-culture at 2 % oxygen tension at day 21 compared to co-culture at 20 % oxygen tension. Co-culture enhanced protein expression of collagen type I, collagen type III, scleraxis, thrombospondin 4 and tenomodulin compared to AD-MSCs alone. Co-culture at 2 % oxygen tension further augmented those genes compared to co-culture at 20 % oxygen tension. Culture at 2 % oxygen tension enhanced mRNA level and protein level of HIF-1α compared to cultures at 20 % oxygen tension. Co-culture at 2 % oxygen tension and with HIF-1α stabilisation using Roxadustat further increased protein levels of collagen type I, collagen type III, scleraxis, thrombospondin 4 and tenomodulin compared to co-culture at 2 % oxygen tension only. Similarly, mRNA level and protein of HIF-1α were augmented using Roxadustat compared to co-culture at 2 % oxygen tension only [79].
**Direct, rabbit cells** BM-MSCs (passage 1) Semitendinosus tendon TCs (passage 1) Ratio BM-MSCs to TCS: 1 to 1 Cells were stretched with 10 % elongation magnitude for 48 h at a frequency of 10 cycles/minute, each cycle consisting of 2 sec of stretch and 2 sec of relaxation	mRNA levels of COLII were not affected in mechanically stretched co-culture cells compared to non-mechanically stretched cells. Co-culture combined with mechanical stretching up-regulated mRNA levels of COLI, COLIII, ALP, OPN, TNC and TNMD compared to not stretched co-culture. Cell metabolism in co-culture was increased under mechanical stimulation compared to co-culture with no mechanical stimulation [82].

Abbreviations: ACAN: aggrecan, AD-MSCs: adipose derived mesenchymal stromal cells, ALP: alkaline phosphatase, ANGPT2: angiopoeitin 2, BM: basal medium, BM-MSCs: bone marrow mesenchymal stromal cells, COL: collagen, COMP: cartilage oligomeric matrix protein, DCN: decorin, EMF: external magnetic field, HIF-1α: hypoxia inducible factor, HUVECs: human umbilical vein endothelial cells, MB-MSCs: menstrual blood mesenchymal stromal cells, mRNA: messenger ribonucleic acid, OPN: osteopontin, PCL: polycaprolactone, PLA: polylactic acid, PRP: platelet rich plasma, RUNX2: runt-related transcription factor 2, SCX: scleraxis, TCs: tendon cells, TGF: transforming growth factor, THBS4: thrombospondin 4, TNAP: tissue non-specific alkaline phosphatase, TNC: tenascin, TNMD: tenomodulin, VEGFA: vascular endothelial growth factor A.

With respect to preclinical assessment, although co-cultures with MSCs have shown promise in several clinical indications (e.g. skeletal myoblasts and MSCs in a cardiac model [88], islets and MSCs in a diabetic model [89], vascular endothelial cells and MSCs in a periodontal model [90], endothelial progenitor cells and MSCs in an ectopic bone model and in a bone model [91]), no co-cultured TCs / MSCs have been transplanted as yet. Instead, there is only one study using rat BM-MSCs and rat patellar tendon stem cells (at ratios 1 to 0, 20 to 1, 10 to 1, 5 to 1, 1 to 1 and 0 to 1); in vitro, the 1 to 1 ratio induced the highest gene expression of TNMD, SCX, TNC, DCN and COLI and in vivo, in a rat patellar tendon defect model, the 1 to 1 ratio cell sheets and the normal tendon had the lowest tendon healing score (the lowest the score the optimal the healing quality) and the highest ultimate stress and Young's modulus values [63]. Despite these very good preliminary data, one cannot but note some major limitations (e.g. the cells were obtained from young and healthy animals; the cells were cultured for 3 weeks; and the defect was 1 mm wide) that place the study rather away from the clinical reality.

## 5 Conclusions

TCs and BM-MSCs are the most studied cell populations in tendon engineering. Yet again, scientific knowledge gained from researcher endeavours has not been translated into clinical and commercial reality. In general, the field recognises as major roadblocks the low number of limited functionality and of poor in vitro expansion capacity TCs and the possibility of heterotopic bone formation of naïve MSCs. In this context, MSC priming towards tenogenic lineage using TC may be a valuable alternative. Admittedly, this line of investigation is still at primitive stage with numerous unanswered or inconclusively answered questions (e.g. cell ratio, direct or indirect culture, time in co-culture required, superiority of the co-culture over traditional differentiation avenues based on media supplements and/or mechanical stimulation and/or scaffold induced signals, appropriate preclinical data, regulatory pathway for the primed cells, etc.). We feel that data obtained to-date clearly demonstrate the potential of the approach for clinical indications with limited availability of permanently differentiated cell populations.

## CRediT authorship contribution statement

**Salomé Guillaumin:** Writing – original draft. **Andrea Rossoni:** Writing – original draft. **Dimitrios Zeugolis:** Writing – review & editing, Visualization, Supervision, Resources, Funding acquisition, Conceptualization.

## Declaration of competing interest

The authors declare that they have no known competing financial interests or personal relationships that could have appeared to influence the work reported in this paper.

## Data Availability

No data was used for the research described in the article.
